# Visual Aspects of Reading Performance in Myalgic Encephalomyelitis (ME)

**DOI:** 10.3389/fpsyg.2018.01468

**Published:** 2018-08-17

**Authors:** Rachel L. Wilson, Kevin B. Paterson, Victoria McGowan, Claire V. Hutchinson

**Affiliations:** Department of Neuroscience, Psychology and Behaviour, College of Life Sciences, University of Leicester, Leicester, United Kingdom

**Keywords:** myalgic encephalomyelitis, chronic fatigue syndrome, reading speed, reading acuity, visual acuity, crowded acuity

## Abstract

People with Myalgic Encephalomyelitis/Chronic Fatigue Syndrome (ME/CFS) report vision-related reading difficulty, although this has not been demonstrated objectively. Accordingly, we assessed reading speed and acuity, including crowded acuity and acuity for isolated words using standardized tests of reading and vision, in 27 ME/CFS patients and matched controls. We found that the ME/CFS group exhibited slower maximum reading speed, and had poorer crowded acuity than controls. Moreover, crowded acuity was significantly associated with maximum reading speed, indicating that patients who were more susceptible to visual crowding read more slowly. These findings suggest vision-related reading difficulty belongs to a class of measureable symptoms for ME/CFS patients.

## Introduction

Myalgic Encephalomyelitis/Chronic Fatigue Syndrome (ME/CFS) is a debilitating disorder, affecting over 250,000 people in the United Kingdom. It represents a substantial disease burden on sufferers, their families, the health service and economy. Marked by debilitating fatigue, it is not well-understood and its diagnosis is controversial. There is no established cause, no test to determine its presence and no definitive outward signs that set it apart from other disorders. In the main, clinicians must rely on patients’ self-perceptions and reports. Although, there are some symptoms, such as those related to cognition that could be quantified using experimental measures, cognitive dysfunction is also a recognized feature of depression, as are many other ME/CFS-reported symptoms such as fatigue, malaise and aching joints/muscles. The result is that those with ME/CFS are frequently incorrectly diagnosed with depressive disorders and, consequently, may receive long periods of inappropriate and unnecessary treatment (Griffith and Zarrouf, 2008). As such, identifying distinct measureable features of ME/CFS is an important issue.

People with ME/CFS often report that they experience difficulty with tasks that rely on visual input (Potaznick and Kozol, 1992; Leslie, 1997; Vedelago, 1997; Hutchinson et al., 2014; Loew et al., 2014). A commonly-identified problem in this context is that of reading where patients report that they find reading difficult and suffer from vision-related symptoms (e.g., pattern glare, headaches, difficulty tracking lines of text) when they read, particularly when reading for prolonged periods of time (Vedelago, 1997; Loew et al., 2014). Here, in the first study of its type, we quantified the impact of ME/CFS on reading using standardized tests of reading speed. We also explored the possible contribution of poor visual acuity to reading-related difficulties ME/CFS, given its fundamental role in the ability to accurately resolve letters and words. Because some ME-related cognitive difficulties have also been reported (see Cockshell and Mathias, 2013 for a review), we also sought to rule out the contribution of higher-level cognitive problems related to language knowledge or ability by measuring word knowledge and verbal concept, given their role in cognitive aspects of reading.

Specifically, in a group of ME patents and matched controls, we compared maximum and average reading speeds, reading acuity (the smallest text size at which participants can read accurately), near visual acuity for isolated words and letters, and crowded visual acuity [the accuracy with which participants can identify a target letter (optotype) surrounded by flanking stimuli]. Word knowledge and verbal concept were assessed as measures of higher-level cognitive aspects of reading. On the basis of previous reports of visual and reading difficulties associated with ME/CFS, our principal expectation that the ME/CFS group might read more slowly than controls.

## Materials and Methods

### Participants

Twenty seven ME/CFS patients and 27 matched controls took part in the study. Controls were age-matched to within ±6 months of the ME/CFS participants. The ME group ranged in age from 17 to 68 years (Mean = 43.5; *SD* = 11.97). The control group ranged in age from 18 to 68 years (Mean = 44.7 years; *SD* = 12.26). Participants were also matched for gender. In each group, there were 23 females and 4 males. All participants had at least completed secondary education to age 18. All patients had an ME/CFS diagnosis of at least 2 years duration, confirmed with the DePaul Symptom Questionnaire (Jason et al., 2010). Only participants who fulfilled these criteria were included. Participants had no history of ocular disease. Before admission to the study, all participants performed acuity tests at near and distance to ensure that any findings in the reading tests were not due to visual impairment. All had normal or corrected-to-normal vision and there were no significant differences between groups for letter acuity at distance or near. Participants confirmed that they did not have a diagnosis of dyslexia. Ethical approval was granted by the University of Leicester. All experimental methods adhered to the tenets of the Declaration of Helsinki. Informed consent was obtained before the study commenced.

### Experimental Measures

Participants were informed that they should use corrected vision for all visual tests and all measures were performed under binocular viewing conditions. Reading performance was determined using 2 standardized tests of reading; The Minnesota Reading Acuity Chart (Mansfield et al., 1993) and Radner Rate of Reading Chart (Radner et al., 1998). Both provide reliable measurements of reading ability in normal and visually impaired individuals (Mansfield et al., 1994). MN Read Acuity Charts are regularly used in clinical practice and have been used to evaluate reading ability in vision-related conditions such as glaucoma (Ishii et al., 2013) and age-related macular degeneration (Patel et al., 2011). Radner Rate of Reading Charts have been used in the clinical assessment of reading performance of low vision patients (Burggraaff et al., 2010). Participants read (out loud) the words on the charts binocularly at a viewing distance of 40 cm.

The MN Read Acuity Chart yielded three performance measures: reading acuity (the smallest print size at which the participant could read without significant errors), maximum reading speed (the reading speed when performance is not limited by print size), and critical print size (the smallest print size at which patients can read with their maximum speed). These were calculated in accordance with the MN Read Acuity Chart (© 1994) instructions. The Radner Rate of Reading Chart yielded four performance measures: reading acuity, maximum reading speed (the greatest number of words read per minute), average reading speed (mean reading speed of all sentences) and critical print size (the smallest print size read at maximum reading speed). All were calculated in accordance with the manufacturer’s instructions.

Single and crowded letter acuity were determined using the Keeler logMar Crowded Test (McGraw and Winn, 1993; Simmers et al., 1999). Near visual acuity for isolated words was determined using The Institute of Optometry Near Card Test (Evans and Wilkins, 2001). To ensure that any findings were not confounded by higher-level cognitive difficulties related to word or language knowledge or ability, all participants completed the Wechsler Adult Intelligence Scale (WAIS) Vocabulary Subtest.

### Statistical Analysis

Matched samples *t*-tests were applied to determine between-group differences. To address the potential issue of family-wise errors, alpha levels required for significant group differences in reading performance were adjusted using Bonferroni correction for multiple comparisons (Bland and Altman, 1995). To determine the relationships between experimental variables linear regression analyses were applied to data from ME/CFS patients, where appropriate. All reported *p*-values are two-tailed.

## Results

**FIGURE 1:**
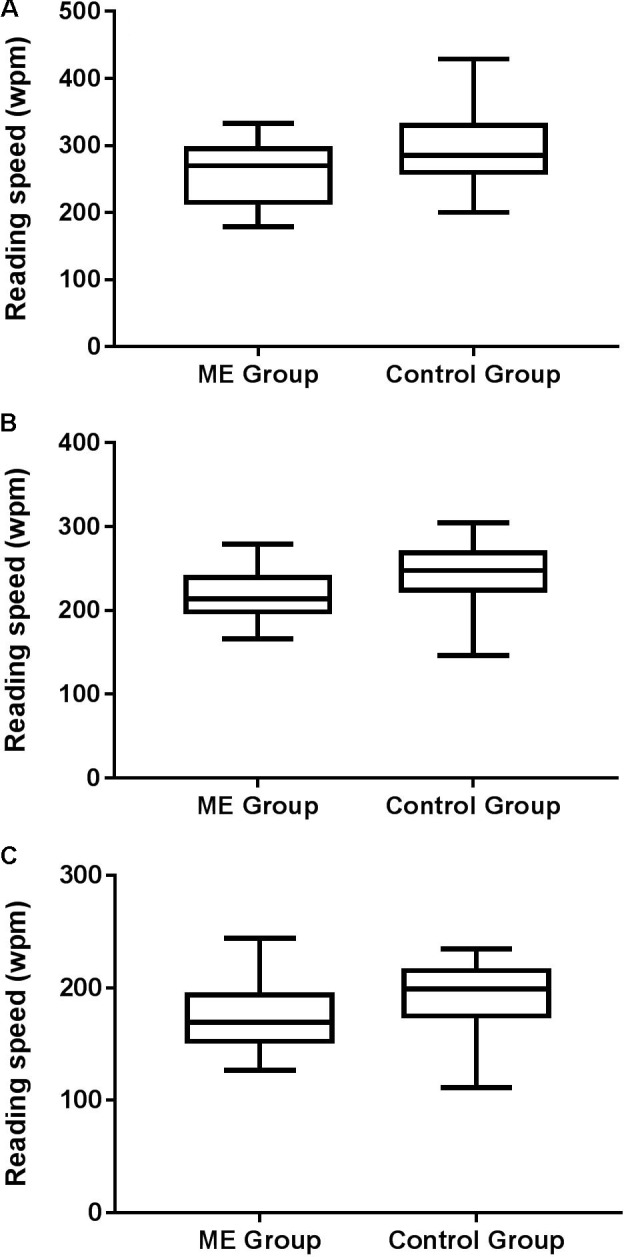
Box and whisker plots showing minimum, 1st quartile, median, 3rd quartile and maximum reading performance (words per minute: wpm) for patients and controls as determined by **(A)** MN Read Acuity Chart maximum reading speed, **(B)** Radner Rate of Reading Chart maximum reading speed, and **(C)** Radner Rate of Reading Chart average reading speed. *Note different y-axis scales on each plot. After correction for multiple comparisons (three measures of reading performance), the alpha level required for a statistically significant difference between groups was p* < 0.017.

Maximum reading speed, as determined by the MN Read Acuity Chart, showed that patients read more slowly than controls [*t*(26) = 2.570, *p* = 0.016; *d* = 0.49] in that they were able to read fewer words per minute. Maximum reading speed, as determined by the Radner Rate of Reading Chart was slower in patients than controls [*t*(26) = 2.906; *p* = 0.007; *d* = 0.55]. Patients’ average reading speed on the Radner Rate of Reading Chart was also slower than controls’ [*t*(26) = 2.125, *p* = 0.043; *d* = 0.41], although this was not significant when corrected for multiple comparisons (**Figure 1**). There were no significant group (patients vs. controls) differences in reading acuity [MN Read Acuity Chart: *t*(26) = 0.950, *p* = 0.351; Radner Rate of Reading Chart: *t*(26) = 1.60, *p* = 0.122] or critical print size [MN Read Acuity Chart: *t*(26) = 0.238, *p* = 0.814; Radner Rate of Reading Chart: *t*(26) = 0.296, *p* = 0.769] (**Table 1**).

**Table 1:** Mean (± 1 SEM) reading acuity and critical print size for patients and controls.

	MN read acuity chart		Radner rate of reading chart	
	Reading acuity	Critical print size	Reading acuity	Critical print size
Patients	-0.03 (0.03)	0.05 (0.03)	0.04 (0.05)	0.15 (0.03)
Controls	-0.06 (0.02)	0.06 (0.02)	-0.03 (0.02)	0.15 (0.03)

*Data for the MN Read Acuity Chart (values shown in logMar) and the Radner Rate of Reading Chart (values shown in logRad) are shown*.

**FIGURE 2:**
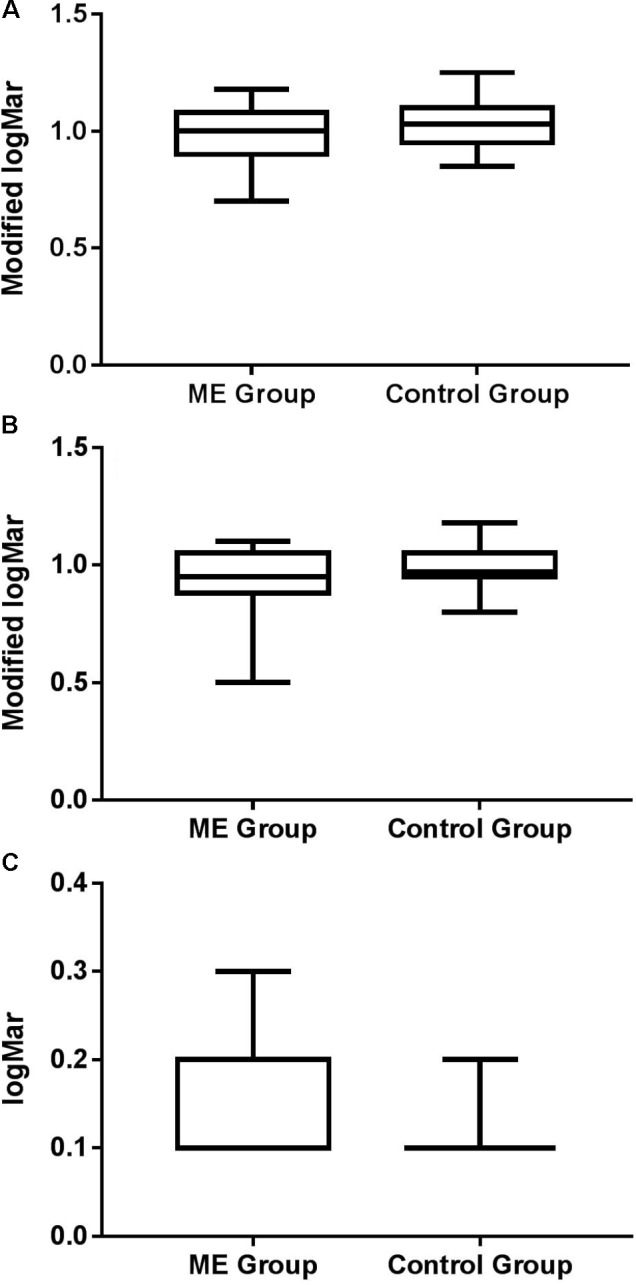
Box and whisker plots showing minimum, 1st quartile, median, 3rd quartile, and maximum for **(A)** uncrowded and **(B)** crowded acuity for patients and controls, expressed as a modified logMAR score (1-logMAR) (c.f. McGraw and Winn, 1993; Simmers et al., 1999). Crowding ratios (crowded: uncrowded) were 1.35 for patients and 1.01 for controls, where higher values represent increased susceptibility to crowding. **(C)** Acuity for isolated words for each group.

Patient and control performance on the logMar Crowded Test (uncrowded and crowded letter acuity) and the Institute of Optometry Near Card Test (acuity for isolated words) are shown in **Figure 2**. There was no significant difference in uncrowded letter acuity between groups [*t*(26) = 1.734; *p* = 0.095]. Patients were, however, more susceptible to visual crowding than controls [*t*(26) = 2.247; *p* = 0.044; *d* = 0.41]. Visual acuity for isolated words did not differ significantly between patients and controls [*t*(26) = 1.911; *p* = 0.057; *d* = 0.38], although it is of note that it did approach significance. There were no differences between groups on the WAIS vocabulary test performance [ME Group: Mean = 48.00 out of a maximum score of 57, *SD* = 7.97; Controls: Mean = 48.89, *SD* = 6.03; *t*(26) = 0.486; *p* = 0.631].

**FIGURE 3:**
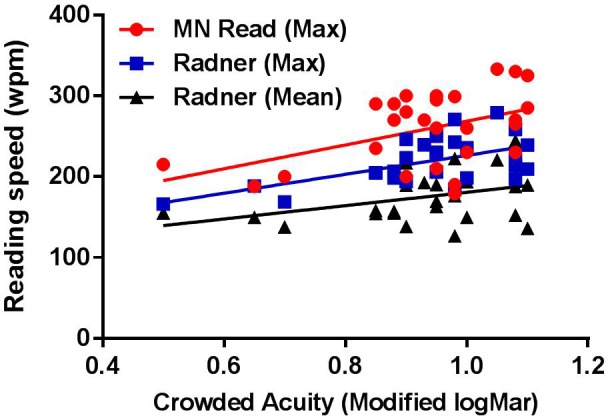
Reading performance [words per minute (wpm)] corresponding to MN Read Acuity Chart maximum reading speed (red circles), Radner Rate of Reading Chart maximum reading speed (blue squares) and Radner Rate of Reading Chart average reading speed (black triangles) plotted against crowded acuity (modified logMar). Lines show best fit linear regression.

The relationships between crowded acuity and each of the reading speed measures are shown in **Figure 3**. Regression analyses showed that crowded acuity significantly predicted maximum reading speed as determined by the MN Read Acuity Chart performance [*R*^2^ = 0.206, *F*(1,26) = 6.798, *p* = 0.017] and the Radner Rate of Reading Chart [*R*^2^ = 0.325, *F*(1,26) = 12.051, *p* = 0.002], but not mean reading speed [*R*^2^ = 0.144, *F*(1,26) = 4.219, *p* = 0.051]. Overall, these results showed that increased susceptibility to visual interference from letters (poor crowded acuity) was associated with slower maximum reading speed (fewer words per minute read). To further determine the relationship between crowded acuity and maximum reading speed, we used robust correlation analysis to correct for possible outliers (Pernet et al., 2013). The results are given in **Table 2**, for which the relationship between crowded acuity scores and maximum reading speed on the Radner Reading Test were most reliably correlated.

**Table 2:** Robust correlation results (r, p, lower, and upper 95% CIs), for the relationship between crowded acuity and maximum reading speed on the MN Read Acuity and Radner Rate of Reading Charts.

	MN read acuity chart	Radner rate of reading chart
Pearson	*r* = 0.454, *p* = 0.017, CI = [0.135, 0.693]	*r* = 0.570, *p* = 0.002, CI = [0.229, 0.774]
Spearman	*r* = 0.309, *p ns*, CI = [–0.089, 0.617]	*r* = 0.456, *p* = 0.016, CI = [0.051, 0.734]
20% bend	*r* = 0.381, *p ns*, CI = [–0.061, 0.676]	*r* = 0.425, *p* = 0.027, CI = [0.045, 0.747]
Skipped Pearson	*r* = 0.454, *p* = 0.017, CI = [0.136, 0.682]	*r* = 0.570, *p* = 0.002, CI = [0.198, 0.769]
Skipped Spearman	*r* = 0.309, *p ns*, CI = [–0.093, 0.629]	*r* = 0.456, *p* = 0.016, CI = [0.071, 0.737]


## Discussion

The findings presented here add to a growing literature demonstrating that vision-related problems and their effects on everyday tasks that involve functional vision (e.g., reading, driving) represent a measurable class of symptoms that are commonly reported by patients with ME/CFS. In the context of the present study, they support the claims of people with ME/CFS that they experience difficulties related to reading, and that visual factors contribute to this phenomenon. ME/CFS patients exhibited slower maximum reading speeds than controls on standardized reading tests. Although reading acuity and acuity for isolated words and letters did not differ significantly between patients and controls, patients were more susceptible to visual crowding. Reading test performance was also correlated with crowded acuity in that patients who read more slowly were more susceptible to the effects of visual crowding. Increased susceptibility to visual crowing was also associated with poor acuity for isolated words. Given that the two groups performed equivalently on the WAIS vocabulary test, our findings are unlikely due to cognitive difficulties related to poor reading performance. In short, our findings suggest that whilst reading problems in ME/CFS are unrelated to poor reading acuity or visual acuity for letters or cognitive deficits, increased susceptibility to visual crowding may be a factor in reading-related difficulties in people with ME/CFS.

Although, there were significant group differences for crowded acuity, there were no significant differences in acuity for isolated words between the ME group and controls, although it is of note that differences between groups did approach significance. This may at first appear counterintuitive. The findings of the present study showed that letter acuity was more susceptible to the effects of visual crowding in people with ME. By extension it would be reasonable to assume that acuity for isolated words would be affected equivalently as a result of the effects of crowding between letters and therefore differences between groups would reach significance. One reason for the absence of a significant difference between groups for isolated word acuity may be due to the test we used, the Institute of Optometry Near Card Test (Evans and Wilkins, 2001). This test may not have been sensitive enough to reveal significant differences between groups. Specifically, the test may suffer from a ceiling effect (best attainable reading acuity is a logMar value of 0.1, corresponding to a decimal acuity value of 0.8).

Deficits in other aspects of binocular vision, such as accommodation or eye movement control may also contribute to reading difficulties in ME/CFS. There is evidence for problems with visual accommodation in ME/CFS, where reduced fusion amplitudes, reduced convergence capacity and a smaller accommodation range have been reported recently (Godts et al., 2016). In the context of reading, poor accommodation has been linked to headaches and visual discomfort in school-age children (Borsting et al., 2003). Similarly, accommodative dysfunction has been linked to poor general reading ability in school-aged children (Shin et al., 2009), although other studies have found little evidence that this is the case (Morad et al., 2002). Follow-up studies examining whether there is a link between poor accommodative function and reading difficulties in ME/CFS are therefore warranted.

Deficits in binocular eye movement control have also been shown previously in non-reading tasks where, compared to controls, patients exhibited impaired anti-saccadic and smooth pursuit eye movements (Badham and Hutchinson, 2013). Studying eye movements while reading may shed light on the causes of reading-related visual discomfort but, to date, no studies have systematically examined eye movements during reading in this group. When we read, our eyes move along each line of text by making a rapid sequence of saccadic eye movements, separated by brief fixational pauses during which visual information is acquired. Studies of this behavior are remarkably informative about moment-to-moment processes in reading and have led to the development of sophisticated models of eye movement control (Reichle et al., 2003). Studying eye-movements during reading may therefore provide a more coherent account of how reading behavior is affected by ME/CFS.

Establishing a fuller picture of the specific aspects of visual and vision-related functions (e.g., reading) affected by ME/CFS could provide valuable insights into visual and even general ME/CFS-related pathology. Furthermore, given the marked impact of vision problems and their functional consequences on everyday quality of life, identifying and treating vision-related symptoms of ME/CFS could provide a means of improving the everyday lives of patients.

## Ethics Statement

This study was carried out in accordance with the recommendations of the Research Ethics Committee, University of Leicester. All subjects gave written informed consent in accordance with the Declaration of Helsinki. The protocol was approved by the School of Psychology Ethics Committee, University of Leicester.

## Author Contributions

CH and KP conceived the study. RW conducted the study with assistance from VM. CH wrote the manuscript with assistance from KP, RW, and VM.

## Conflict of Interest Statement

The authors declare that the research was conducted in the absence of any commercial or financial relationships that could be construed as a potential conflict of interest.
